# SOCS3-Mediated Blockade Reveals Major Contribution of JAK2/STAT5 Signaling Pathway to Lactation and Proliferation of Dairy Cow Mammary Epithelial Cells *in Vitro*

**DOI:** 10.3390/molecules181012987

**Published:** 2013-10-17

**Authors:** Yu-Ling Huang, Feng Zhao, Chao-Chao Luo, Xia Zhang, Yu Si, Zhe Sun, Li Zhang, Qing-Zhang Li, Xue-Jun Gao

**Affiliations:** Key laboratory of Dairy Science of Education Ministry, Northeast Agricultural University, Harbin 150030, Heilongjiang, China; E-Mails: hyllw335474663@163.com. (Y.-L.H.); erjinzhi@126.com (F.Z.); luochaochao839505@163.com (C.-C.L.); zx291765746@126.com (X.Z.); siyu19880329@163.com (Y.S.); sunzhe1998@163.com (Z.S.); zhangli19782000@sohu.com (L.Z.); qingzhangli2004@163.com (Q.-Z.L.)

**Keywords:** SOCS3, dairy cow mammary epithelial cells, milk protein synthesis, milk fat synthesis, lactation

## Abstract

Suppressor of cytokine signaling 3 (SOCS3) is a cytokine-induced negative feedback-loop regulator of cytokine signaling. More and more evidence has proved it to be an inhibitor of signal transducers and activators of transcription 5 (STAT5). Here, we used dairy cow mammary epithelial cells (DCMECs) to analyze the function of SOCS3 and the interaction between SOCS3 and STAT5a. The expression of SOCS3 was found in cytoplasm and nucleus of DCMECs by fluorescent immunostaining. Overexpression and inhibition of SOCS3 brought a remarkable milk protein synthesis change through the regulation of JAK2/STAT5a pathway activity, and SOCS3 expression also decreased SREBP-1c expression and fatty acid synthesis. Inhibited STAT5a activation correlated with reduced SOCS3 expression, which indicated that SOCS3 gene might be one of the targets of STAT5a activation, DCMECs treated with L-methionine (Met) resulted in a decrease of SOCS3 expression. SOCS3 could also decrease cell proliferation and viability by CASY-TT detection. Together, our findings indicate that SOCS3 acts as an inhibitor of JAK2/STAT5a pathway and disturbs fatty acid synthesis by decreasing SREBP-1c expression, which validates its involvement in both milk protein synthesis and fat synthesis. In aggregate, these results reveal that low SOCS3 expression is required for milk synthesis and proliferation of DCMECs *in vitro*.

## 1. Introduction

Suppressor of cytokine signaling (SOCS) proteins are inhibitors of JAK/STAT pathway and act as a classical negative feedback loop that modulates cellular responsiveness to cytokines, first discovered in 1997 by different groups [1,2,3]. As a member of SOCS family, numerous biological functions have been attributed to SOCS3 activity, ranging from growth and development to lactation and apoptosis, from insulin resistance to obesity, as well as immunity and inflammatory response [4,5,6]. SOCS3 expression is stimulated by various cytokines, including growth hormone (GH), prolactin (PRL), insulin (INS), insulin-like growth factor-1 (IGF-1) and inhibited by glucocorticoids [7,8]. The interaction between SOCS3 and STAT5 has been intensively studied recently. SOCS3 has a potent inhibitory effect on STAT5, and its expression is STAT5 dependent in breast cancer cells [9], and appears to be independent from STAT5 activation *in vivo* [10] and not a prominent negative regulator of STAT5 signaling in mouse during pregnancy and lactation [11]. However, in other experiments SOCS3 was demonstrated to inhibit PRL induction of milk protein gene expression and STAT5 activation *in vitro* [12]. Whether SOCS3 is a negative feedback-loop regulator of the JAK2/STAT5 signaling pathway in dairy cow mammary epithelial cells (DCMECs) is not fully known, further experiments are needed to clarify this point. STAT5 was originally identiﬁed as a transcription factor that regulated the β-casein gene in response to PRL. It is encoded by two closely related genes, STAT5a and STAT5b [13]. STAT5 is a major factor controlling expression of milk protein genes and cell proliferation, which has largely been investigated in mouse and bovine [14,15,16,17]. These reports differ from that of Bionaz and Loor *et al*. [18], who supported a minor role of JAK2/STAT5 signaling for milk protein synthesis in bovine mammary gland *in vivo*. The function of STAT5 gene and regulatory networks linking STAT5 in milk production of DCMECs remains controversial. Whether SOCS3 participates in JAK2/STAT5 pathway to regulate milk protein synthesis in DCMECs is unknown.

The mammalian target of rapamycin (mTOR) in promoting protein synthesis has been well described, and sterol regulatory element binding protein (SREBP) plays an important role in regulating lipid synthesis [19]. Whether SOCS3 affects lactation through these pathways in DCMECs and the molecular mechanisms mediate their effects remain largely unknown. Studies have investigated the relationship between SOCS3 and SREBP-1c, but no study has been performed in DCMECs [20]. SOCS3 might be involved in regulation of milk fat synthesis in DCMECs [21]. Understanding the regulatory effects of individual amino acids (AAs) on milk protein synthesis is important for lactation. Methionine (Met) is an essential AA that plays fundamental roles in protein synthesis and a number of cellular processes including cell proliferation [22,23]. In low-amino-acid medium, expression of SOCS3 was increased in Huh-7 cells [24]. While no study has directly shown regulation of SOCS3 expression by Met, the effects of Met on the expression of SOCS3 remain largely elusive. The present study aims to establish direct actions of SOCS3 on lactation and proliferation of DCMECs, and to further elucidate the role of SOCS3-mediated blockade of JAK2/STAT5 signaling pathway.

## 2. Results and Discussion

### 2.1. SOCS3 Overexpression Decreased Cell Proliferation and Negatively Regulated JAK2/STAT5a Signaling Pathway as well as Fatty Acid Synthesis in DCMECs

**Figure 1:**
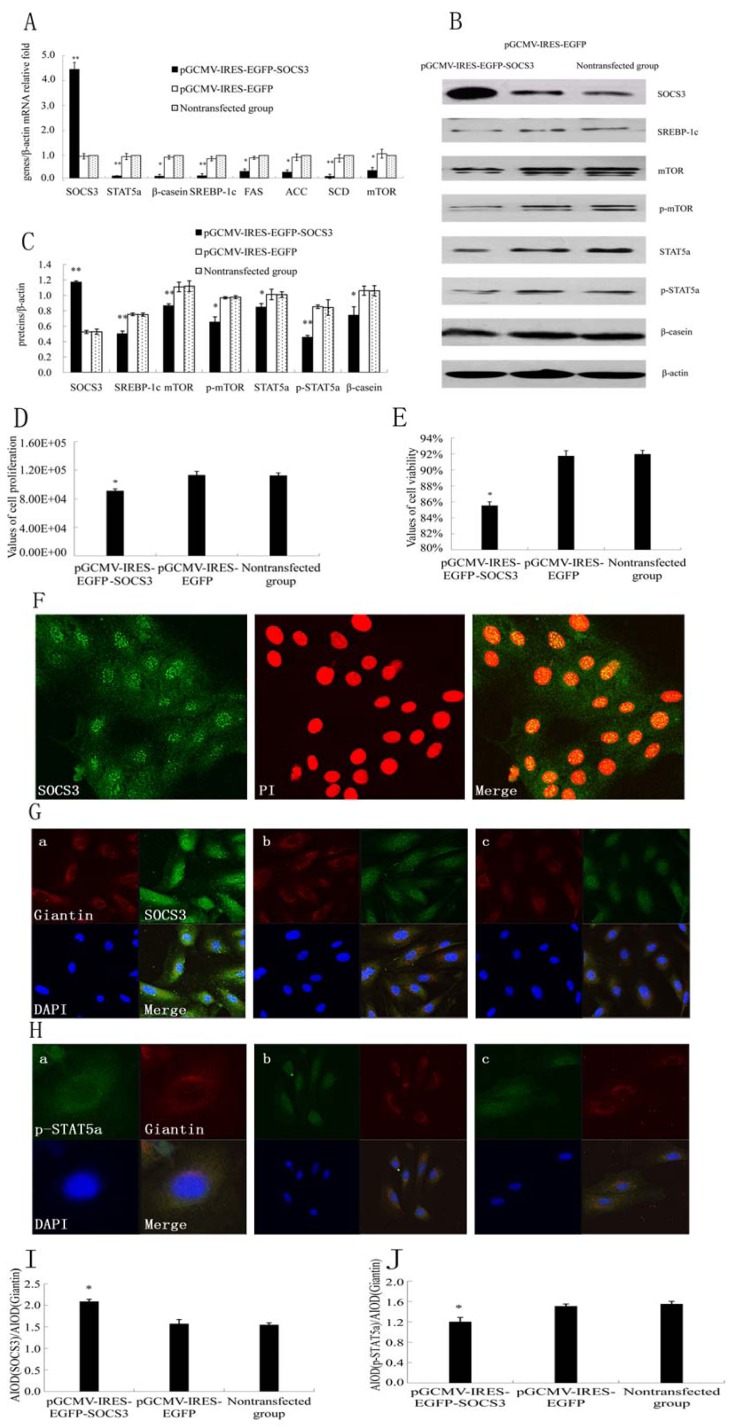
Effect of SOCS3 overexpression on lactation and proliferation in DCMECs. DCMECs were divided into three groups: transfected with a SOCS3 expression construct or empty vector (control group), nontransfected, grew for 48 h. (**A**) Gene expressions in DCMECs growing for 48 h were detected using qRT-PCR and normalized to β-actin mRNA expression; (**B**) Western blotting results of SOCS3, STAT5a, p-STAT5a, mTOR, p-mTOR, SREBP-1c, β-casein and β-actin in DCMECs growing for 48 h after SOCS3 overexpression; (**C**) Results of gray scale scan of SOCS3, STAT5a, p-STAT5a, mTOR, p-mTOR, SREBP-1c and β-casein/β-actin relative fold by western blotting after SOCS3 overexpression; (**D**,**E**) Proliferation and viability of DCMECs growing for 24 h were measured using CASY; (**F**) Localization of SOCS3. SOCS3 (green), nuclei(red). Scale bars = 75 μm. (**G**) Overexpressed SOCS3 proteins had higher levels of nuclear localization of DCMECs. a: Cells were transfected with a SOCS3 expression construct and grew for 24 h; b: Cells were transfected with an empty vector and grew for 24 h; c: Nontransfected cells. SOCS3(green), Giantin(Red) and DAPI(blue). Scale bars = 20 μm; (**H**) Immunofluorescent staining for p-STAT5a was performed on DCMECs. a: Cells were transfected with a SOCS3 expression construct and grew for 24 h; Scale bars = 75 μm; b: Cells were transfected with an empty vector and grew for 24 h ; Scale bars = 20 μm; c: Nontransfected cells. p-STAT5 (green), Giantian (red) and nuclei were counterstained with DAPI (blue). Scale bars = 20 μm; (**I**,**J**) The analysis of fluorescence staining after SOCS3 overexpression. Different superscripts denote signiﬁcant difference relative to controls. * or ** means significant difference compared with negative control (*p <* 0.05 or *p <* 0.01).

SOCS3 is an established negative regulator of STATs. To test whether SOCS3 was able to prevent STAT5a activation in DCMECs, SOCS3 was overexpressed and the expressions of related genes were detected by qRT-PCR and WB at 48 h, respectively. Meanwhile cell proliferation and viability were examined by CASY. SOCS3 mRNA expression was signiﬁcantly increased in pGCMV-IRES-EGFP-SOCS3 groups (Figure 1A). The SOCS3 protein expression in pGCMV-IRES-EGFP-SOCS3 groups was markedly higher than that in pGCMV-IRES-EGFP group (Figure 1B,C). We observed a significant 60%–65% increase of SOCS3 protein in DCMECs. It indicated that SOCS3 was stable–highly expressed in DCMECs infected with pGCMV-IRES-EGFP-SOCS3. The mRNA levels and protein expressions of STAT5a, p-STAT5a, mTOR, p-mTOR and β-casein were obviously reduced in cells transfected with SOCS3 compared with empty vector group (Figure 1A–C). Here we demonstrated that overexpression of SOCS3 in DCMECs signiﬁcantly inhibited cell growth and viability (Figure 1D,E). In the present study, we found that SOCS3 protein was distributed both in the cytoplasm and nucleus in DCMECs. Infrequently, cells displayed a speckled nuclear SOCS3 expression pattern (Figure 1F). In experiments of overexpression of SOCS3 protein, we found that SOCS3 had higher levels of nuclear localization when their levels rose (Figure 1G,I). Additionally, localization of p-STAT5a was assessed by immunofluorescence after SOCS3 overexpression. p-STAT5a protein was distributed both in the cytoplasm and nucleus. After transfection with SOCS3 gene, p-STAT5a expression level was down-regulated in nucleus (Figure 1H,J), which was consistent with the result of WB.

To demonstrate the effect of SOCS3 on lipid synthesis, we detected expressions of key genes on lipid metabolism such as SREBP-1c, fatty acid synthase (FAS), acetyl-CoA carboxylase (ACC) and stearoyl-CoA desaturase (SCD) mRNA levels. Results showed that overexpression of SOCS3 downregulated SREBP-1c, FAS, ACC and SCD mRNA levels (Figure 1A). The expression of SREBP-1c protein by western blotting was at the same trend with mRNA level. SOCS3 evidently inhibited the expression of SREBP-1c protein (Figure 1B,C). These results revealed that overexpression of SOCS3 inhibited SREBP-1c expression and decreased fatty acid synthesis.

### 2.2. Inhibition of SOCS3 Increased the Expressions of Key Genes on Lactation and Proliferation of DCMECs

**Figure 2:**
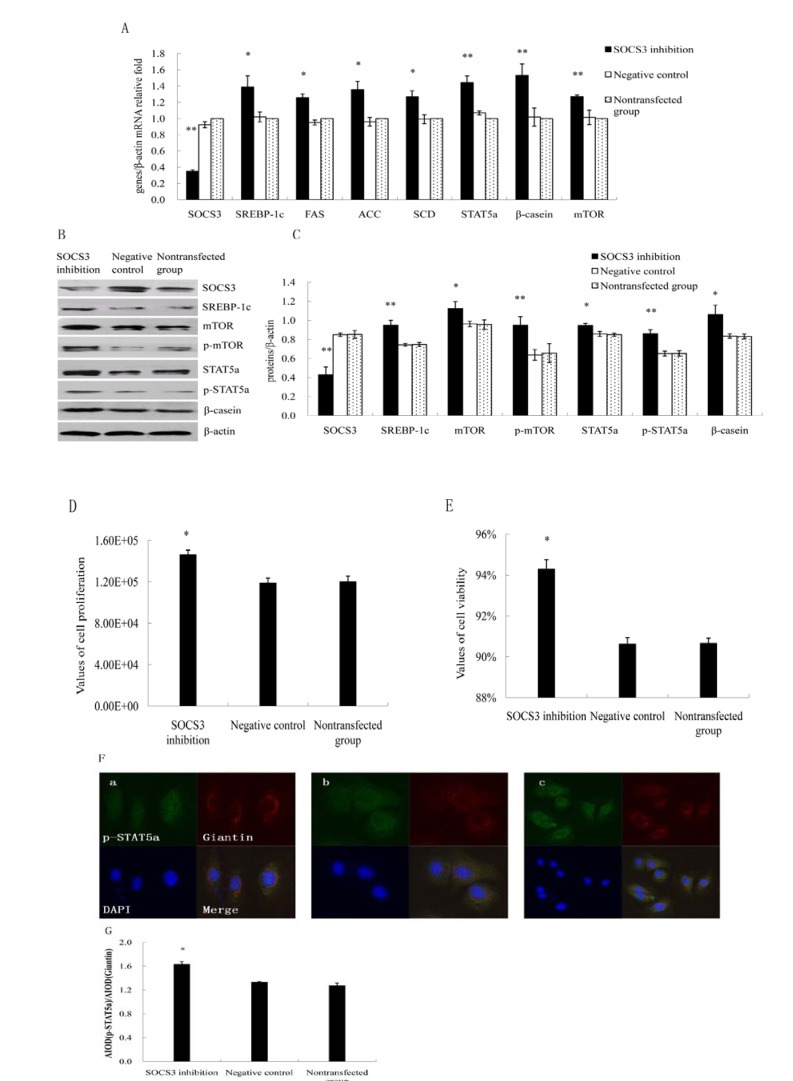
Effect of SOCS3 inhibition on lactation and proliferation of DCMECs. DCMECs were divided into three groups: transfected with a SOCS3 siRNA or negative control (control group), nontransfected, grew for 24 h. (**A**) Relative mRNA expression of different genes in DCMECs growing for 24 h were measured by qRT-PCR after SOCS3 inhibition; (**B**) Western blotting results of SOCS3, STAT5a, p-STAT5a, mTOR, p-mTOR, SREBP-1c, β-casein and β-actin after SOCS3 inhibition in DCMECs growing for 24 h; (**C**) Results of gray scale scan of SOCS3, STAT5a, p-STAT5a, mTOR, p-mTOR, SREBP-1c and β-casein /β-actin relative fold by western blotting after SOCS3 inhibition at 24 h; (**D**,**E**) Proliferation and viability of DCMECs growing for 24 h were measured using CASY; (**F**) Fluorescence staining after SOCS3 inhibition. a: Cells were transfected with SOCS3 siRNA; b: Negative control; c: Nontransfected cells. Scale bars = 75 μm in a and b; 20μm in c; (**G**) The analysis of fluorescence staining after SOCS3 inhibition. * or ** means significant difference compared with negative control group (*p <* 0.05 or *p <* 0.01).

Using siRNA targeted to SOCS3, we examined the effect of SOCS3 silencing on lactation and proliferation in DCMECs. SOCS3 was inhibited and the expressions of certain genes were detected by qRT-PCR and WB at 24 h, respectively. Meanwhile cell proliferation and viability were examined by CASY. SOCS3 knock-down after siRNA transfection was veriﬁed using qRT-PCR. SOCS3 inhibition increased the expression of STAT5a, p-STAT5a, mTOR, p-mTOR, SREBP-1c, FAS, ACC, SCD and β-casein in cells transfected with SOCS3 compared with negative control group (Figure 2A). The mRNA levels and protein expressions of different genes were shown in Figure 2A–C. Silencing of SOCS3 resulted in increased proliferation and viability (Figure 2D,E). Additionally, localization of p-STAT5a was assessed by immunofluorescence after SOCS3 inhibition. p-STAT5a protein expression was up-regulated in nucleus (Figure 2F,G), which was consistent with the result of WB.

### 2.3. STAT5a was Required for SOCS3 Activation in DCMECs

**Figure 3:**
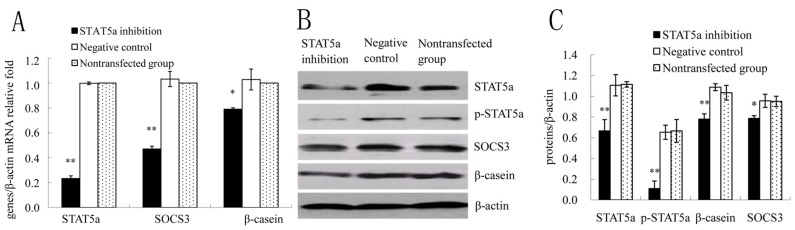
STAT5a was necessary for SOCS3 expression in DCMECs. DCMECs were divided into three groups: transfected with a STAT5a siRNA or negative control (control group), nontransfected, grew for 48 h. (**A**) Relative mRNA levels of STAT5a, SOCS3, β-casein genes in DCMECs growing for 24 h were measured by qRT-PCR after STAT5 inhibition. (**B**) Western blotting analysis of STAT5a, p-STAT5a, SOCS3, β-casein in DCMECs growing for 24 h subjected to STAT5a inhibition. β-actin served as loading control; (**C**) Results of gray scale scan of STAT5a, p-STAT5a, SOCS3 and β-casein/β-actin relative fold by western blotting after STAT5a inhibition at 24 h. * or ** means significant difference compared with negative control (*p <* 0.05 or *p <* 0.01).

It was investigated whether STAT5a played an important role in the mammary gland on the transcriptional regulation of SOCS3. Silencing of STAT5a gene expression was made by transfection with STAT5a siRNA for 24 h. In STAT5a siRNA-treated cells, expression of p-STAT5a was low and difﬁcult to detect (Figure 3A). We assessed the effect of silencing of STAT5a gene expression on SOCS3 in DCMECs, qRT-PCR and WB were used to investigate mRNA levels and protein expressions of STAT5a, p-STAT5a, SOCS3, β-casein, respectively. Data showed that they were all downregulated in STAT5a inhibition group compared with negative control group (Figure 3B–D), suggesting that this transcription factor was responsible for SOCS3 expression in DCMECs.

### 2.4. The Effect of Met on SOCS3 to Regulate β-Casein Expression and Cell Proliferation

**Figure 4:**
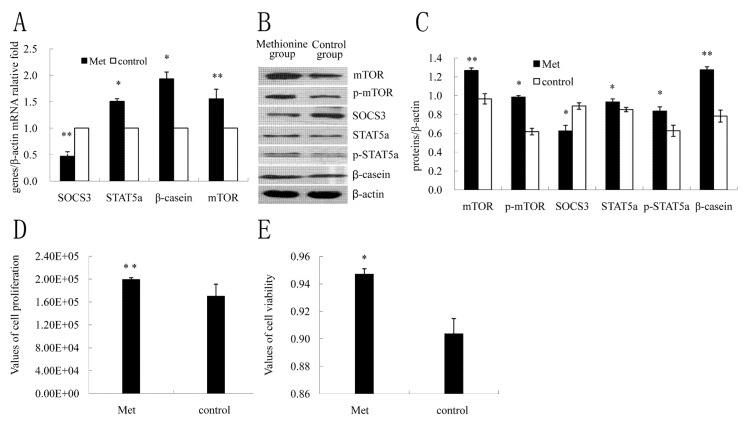
Effect of Met on SOCS3 to regulate β-casein expression and cell proliferation in DCMECs. DCMECs were treated with Met (0.6 mmol L^−1^) for 24 h, not treated (control). (**A**) Changes of mRNA levels of key genes on milk protein synthesis were detected by qRT-PCR; (**B**) Western blotting results of SOCS3, STAT5a, p-STAT5a, mTOR, p-mTOR, β-casein and β-actin at 24 h after treatment with Met; (**C**) Results of gray scale scan of SOCS3, STAT5a, p-STAT5a, mTOR, p-mTOR, β-casein/ β-actin relative fold by western blotting after treatment with Met; (**D**,**E**) Cell proliferation and viability were determined by the CASY assay. Different superscripts denote signiﬁcant differences relative to controls. * or ** means significant difference compared with negative control (*p <* 0.05 or *p <* 0.01).

To determine the effect of Met on β-casein expression and cell proliferation in DCMECs, the cells were treated with Met (0.6 mmol L^−1^) and harvested at 24 h after treatment, according to our laboratory’s previous description [25]. Cell viability and proliferation were determined with CASY. The mRNA levels and protein expressions of SOCS3, STAT5a, p-STAT5a, mTOR, p-mTOR and β-casein were assessed by qRT-PCR and western blotting, respectively. Treatment with Met resulted in markedly increasing of mRNA levels of mTOR, STAT5a, β-casein (Figure 4A) as well as protein expressions of STAT5a and p-STAT5a, mTOR and p-mTOR, β-casein (Figure 4B,C), whereas the mRNA level and protein expression of SOCS3 were both decreased (Figure 4B,C). Results also showed that in comparison to the control group, cell viability and proliferation significantly were increased (Figure 4D,E). These data indicated that the expression of SOCS3 was inhibited by Met.

### 2.5. Discussion

By the experiments of overexpression and inhibition of SOCS3, we demonstrated that SOCS3 inhibited β-casein gene expression and cell proliferation as an inhibitor of JAK2/STAT5a signaling pathway in DCMECs. The relative level of STAT5a transcript and the protein expressions of STAT5a and p-STAT5a in transfected cells correlated with expression of β-casein in DCMECs. More recent studies in mice and human provide a direct link between the SOCS3 and STAT5 [26,27]. SOCS3 serves as a negative regulator of JAK-STAT signalling via several distinct mechanisms, but there is no study reporting the negative feedback mechanism through its direct interaction with the STAT5 protein, though the general trend of their results indicates a role of SOCS3 in attenuating STAT5 expression [28,29]. Together, these data indicate that SOCS3, presumably through the inhibition of STAT5a activation, is an inhibitor of protein synthesis in DCMECs.

The role of STAT5a/b in mammary gland has been controversially discussed, with conﬂicting evidence depending on the model studied. However, in mouse and bovine mammary gland, increasing evidence suggest that STAT5a is crucial for mammary development and milk protein expression [30,31]. Thus, we investigated whether STAT5a played the same role in DCMECs. The inhibition of STAT5a resulted in decrease of mRNA level and protein expression of β-casein. This result is consistent with previous studies that STAT5 deficiency in mice mammary gland leads to a near-complete loss of lobuloalveolar development, a reduction in expression of milk protein genes during pregnancy and lactation failure [32,33]. Based on these findings, it is concluded that SOCS3 participates in JAK2-STAT5a pathway to regulate milk protein synthesis in DCMECs.

To address the mechanism of interaction between STAT5a and SOCS3, we sought to determine if overexpressing and inhibiting SOCS3 proteins changed activated STAT5a protein levels in a manner that was dependent on SOCS3 localization, we used immunofluorescence to observe the localization of SOCS3, we found that SOCS3 protein was distributed both in the cytoplasm and nucleus and overexpressed SOCS3 protein primarily localized in the nucleus where they might reduce the activity of STAT5a protein in DCMECs. The level of p-STAT5a increased in the nucleus after inhibition of SOCS3. Our results suggest that the expression and subcellular localization of p-STAT5a are regulated by SOCS3, which would provide an evaluation at the cellular level by which SOCS3 inhibits STAT5a activity. Therefore, these results are consistent with the Kyeong-Hee Lee who found the translocation of SOCS3 from the cytoplasm to the nucleus under certain biological conditions [34], thus we predict that there is a possible mechanism that a protein such as STAT5a interacted with SOCS3 might bring it into the nucleus. 

Here, SOCS3 expression in DCMECs was dependent on STAT5a, as evidenced by the lack of SOCS3 expression in DCMECs with siRNA targeted to STAT5a. Considering with other reports, we deduced that SOCS3 genes might be one of STAT5a activation targets. This result was consistent with the report of Ehrentraut S *et al*. that inhibition of STAT5 activation by treatment with the STAT5 inhibitor pimozide was accompanied by dose-dependent losses of SOCS2/3 expression in all three PCM1-JAK2 cell lines (human leukemia/lymphoma cell lines), MAC-1/2A/2B [35]. This result differed from that of Le Provost *et al*. [36], who found STAT5a was not involved in the stimulation of the expression of SOCS3 in virgin mice mamary gland tissue. To determine whether SOCS3 expression through STAT5a, it is essential to identify the STAT5-binding sites in the SOCS3 gene promoter. STAT5 could be recruited to the STAT-response element (pSRE) of the SOCS3 promoter to initiate transcription of it in human and sequence analysis of the mouse SOCS3 promoter region revealed STAT5 binding site [9,37]. According to these, we inferred that activated STAT5a might bind to the promoter of SOCS3 in DCMECs. To sum up, SOCS3 is a negative feedback-loop regulator of the JAK2/STAT5a signaling pathway in DCMECs. Briefly, STAT5a increases SOCS3 expression, and then SOCS3 in turn acts to limit further STAT5a signaling, thus it is realized that they complete an intracellular negative feedback loop. This is the first report that SOCS3 inhibits milk protein synthesis in DCMECs. Up to date, the function and mechanism of SOCS3 in milk protein synthesis is far more established.

Recent studies in rodents and ruminants have highlighted a role of mTOR in the regulation of milk protein synthesis [38,39]. In this study we also found SOCS3 inhibited the expression and phosphorylation of mTOR. This negative effect of SOCS3 does not appear to be DCMECs specific because it has been described recently also in murine models of oxygen-induced retinopathy and cancer [40]. This result further revealed that SOCS3 involved in regulation of milk synthesis in DCMECs. The precise mechanism by which SOCS3 regulates mTOR remains to be elucidated.

The number and activity of DCEMCs are determining factors for milk production [41]. Thus, we next elucidated the effects of SOCS3 on cell proliferation. By experiments with CASY, we found overexpression of SOCS3 inhibited cellular growth, whereas SOCS3 inhibition induced cellular growth. Our data suggest that SOCS3 itself acts as an inhibitor of cell proliferation. Furthermore, SOCS3 has a role as negative regulator of STAT5a or mTOR phosphorylation. These results suggest that SOCS3 might inhibit cellular proliferation at least via a STAT5a or mTOR independent mechanism. This finding is similar to studies carried out in human lung cancer where SOCS3 also yielded a decrease of cellular growth [42].

Here, our results verify that SOCS3 affects SREBP-1c expression and its downstream regulatory targets, FAS, SCD and ACC in DCMECs. This result contrasts markedly with earlier studies, in insulin signaling SOCS3 overexpression increased SREBP-1c and its downstream regulatory targets in livers of morbidly obese women and obese diabetic mice [43,44]. It is unclear whether the binding between SREBP-1c and SOCS3 is direct or through expressions of key genes on milk synthesis such as mTOR or STAT5a. SREBP-1 plays an important role in integrated regulation of lipid synthesis in bovine [45]. The discovery of a connection between SOCS3 and SREBP-1c opens a new chapter in our understanding of the molecular mechanisms regulating lipid synthesis. 

Met is an essential amino acid that serves as a substrate for protein synthesis. Here, treatment with Met resulted in more cell proliferation and marked increase of expression of mTOR, STAT5a and β-casein. Surprisingly, the abundance of SOCS3 did not increase but actually decreased. The presence of essential AAs led to directly induced mTOR activation such as leucyl-tRNA synthetase (LRS) directly bound to Rag GTPase to activate mTORC1 [46], and contributed to enhancement of milk protein gene expression [47]. We inferred that the positive effect of Met on mTOR activation would be the main reason to decrease SOCS3 expression. But the mechanism is not known. Combined with our report, we predict that maintaining SOCS3 in a low level state is important to ensure lactation and proliferation of mammary epithelium instead of initiating apoptosis. Met can up-regulate milk protein synthesis partly via the inhibition of expression of SOCS3 expression.

## 3. Experimental

### 3.1. Cell Preparation and Treatments

DCMECs were cultured according to the previous method of our laboratory reported by Lu *et al*. [48]. The cells were cultured in Dulbecco’s modiﬁed Eagle medium: Nutrient Mixture 12 (DMEM: F12) supplemented with 10% fetal bovine serum (FBS), insulin (bovine, 5 μg mL^−1^), hydrocortisone (1 μg mL^−1^), penicillin (100 U mL^−1^) and streptomycin (0.1 mg mL^−1^). Prior to the experimental treatments, cells were plated at 3 × 10^4^ cells cm^−2^ in six-well plates, and the medium was replaced with DMEM: F12 containing insulin (5 μg mL^−1^), hydrocortisone (1 μg mL^−1^), and prolactin (ovine, 3 μg mL^−1^) without FBS. 

### 3.2. Cell Viability Assay

Cell viability was determined with a CASY TT Analyser System (Schärfe System GmbH, Reutlingen, Germany) according to the manufacturer’s instructions. After calibration with dead and vital DCMECs cells, cursor positions were set to 11.75 to 50.00 μm (evaluation cursor) and 7.63 to 50.00 μm (standardization cursor). Cells were trypsinized. The cells diluted with CASY electrolyte solution (1:100) was examined using CASY-TT, 100 μL aliquots were analyzed in triplicate.

### 3.3. RNA Extraction and Quantitative Real-Time PCR

Total RNA extraction of DCMECs was performed using Trizol reagent (Invitrogen, Carlsbad, CA, USA). Subsequently, integrity and quality of RNA were quantified by gel analysis and reverse transcribed into cDNA using thermoscript reverse transcriptase (TaKaRa, Dalian, China) according to the manufacturer’s protocol. Quantitative real-time PCR reactions were performed using real-time PCR Kit Sensimix TM SYBR&Flurescen, and the analysis was performed by an ABI PRISM 7300 RT-PCR System (Applied Biosystems, Foster City, CA, USA) in a total volume of 20 μL using 96-well microwell plates. All the following gene mRNA were normalized to β-actin mRNA level. The primers of these gene transcripts were as follows: SOCS3: sense5'-GAGAAGATCCCTCTGGTGTTGA-3', antisense5'-GGTCCAGGAACTCCCGAAT-3'; STAT5a: sense5'-GTCCCTTCCCGTGGTTGT-3', antisense5'-CGGCCTTGAATTTCATGTTG-3'; β-casein: sense5'-AACAGCCTCCCACAAAAC-3', antisense5'-AGCCATAGCCTCCTTCAC-3; SREBP-1c: sense5'-AGTAGCAGCGGTGGAAGT-3', antisense5'-GCAGCGGCTCTGGATT-3'; mTOR: sense5'-ATGCTGTCCCTGGTCCTTATG-3', antisense5'-GGGTCAGAGAGTGGCCTTCAA-3'; FAS: sense5'-CCACGGCTGTCGGTAAT-3', antisense5'-CGCTCCCACTCATCCTG-3'; ACC: sense5'-AGACAAACAGGGACCATT-3', antisense5'-AGGGACTGCCGAAACAT-3'; SCD: sense5'-CTGTGGAGTCACCGAACC-3', antisense5'-TAGCGTGGAACCCTTTT-3'; β-actin: sense5'-AAGGACCTCTACGCCAACACG-3', antisense5'-TTTGCGGTGGACGATGGAG-3'. All RT-PCR reactions were performed according to the previous report [49]. RT-PCR analysis was performed by the comparative CT method. 

### 3.4. Immunofluorescence

The DCMECs were seeded on glass coverslips to 30-50% conﬂuency in six-well plates. They were then transiently transfected with pGCMV-IRES-EGFP-SOCS3 expression vector or siRNA targeted to SOCS3 and incubated for 24 h, then cells were washed twice with PBS and ﬁxed in 4% (*w/v*) ice-cold paraformaldehyde at 4 °C for 10 min. Next, fixed cells were blocked in blocking buffer (in tris-buffered saline with 5% BSA and 0.1% TritonX-100) for 1 h at 37 °C, then incubated with either anti-SOCS3 primary antibody or anti-p-STAT5a primary antibody at 1:50 dilution for 1 h at 37 °C. After washing three times in TBS/T, specimens were incubated in the dark with FITC-conjugated anti-rabbit IgG at 1:200 dilution for 1 h at 37 °C and DAPI for 10 min at 37 °C. Finally, after washing three times in TBS/T, the coverslips were mounted onto confocal microscopy with a Leica TCS-SP2 AOBS confocal microscope to detect SOCS3, p-STAT5a expression, respectively.

### 3.5. Western Blot Analysis

Western blot analysis was performed using standard techniques reported by LiMin Lu [48]. Total cell lysate containing about 30 μg protein was separated on 10% SDS-PAGE gel and transferred onto nitrocellulose membranes (Bio-RAD, Shanghai, China). Membranes were blocked in 5% skim milk (in tris-buffered saline with 5% skim milk and 0.1% Tween-20). Membranes were probed with primary antibodies specific for the following antibodies: SOCS3(H-103; Santa Cruz Biotechnology Inc., Santa Cruz, CA, USA), STAT5a, p-STAT5a, mTOR, p-mTOR (Cell Signaling Technology, Beverly, MA, USA), SREBP-1c (the mature form, 68kD) (Santa Cruz), β-casein (Abbiotec, USA) and β-actin (Santa Cruz), followed by a second incubation with secondary antibodies [1:1000] conjugated to HRP (ZSGB-BIO, Beijing, China). The chemiluminescence detection of HRP-conjugated secondary antibodies was performed using Super ECL plus (ApplyGEN, Beijing, China).

### 3.6. Construction of SOCS3 Eukaryotic Expression Plasmid and Transfection

Total RNA from cultured DCMECs was extracted using Trizol reagent (Invitrogen), The cDNA was synthesized and the PCR product was inserted into pMD18-T plasmids (TaKaRa Company), followed by identifying with restriction enzyme *Eco*R I and *Sal* I (TaKaRa Company) and DNA sequencing. The SOCS3 gene was cloned into pGCMV-IRES-EGFP vector (GenePharma Co. Ltd, Shanghai, China). Therefore combinant plasmids were obtained and identiﬁed by digestion with *Eco*R I and *Sal* I. The following primers were designed with particular restriction enzyme sites to clone the complete coding region of SOCS3. Forward primer 5'-CGGAATTCATGGTCACCCACAGCAAGTT-3' (*Eco*R I) and reverse primer 5'-ACGCGTCGACCTAAAGCGGGGCATCGTACT-3' (*Sal* I). The optimized ampliﬁcation conditions were annealing at 58.9 °C and extension at 72 °C for 35 cycles.

Transient transfections was according to the previous report of Lu [48]. Briefly, DCMECs were transfected with pGCMV-IRES-EGFP-SOCS3 or empty vectors which was added to balance the total amounts of transfected DNA samples using Lipofectamine TM 2000 (LF2000, Invitrogen,) according to the manufacturer’s protocol. Nontransfected cells were conducted as controls in the same way of transfection. Cells were cultivated for 48 h. The cells were then collected for further experiments.

### 3.7. Small Interfering RNA Transfection

Small interfering RNA (transfection SOCS3 or STAT5a siRNA, GenePharma Co., Ltd.) and negative control RNA-oligonucleotides (GenePharma Co., Ltd.). DCMECs were transfected with the SOCS3 or STAT5a siRNA and negative control using Lipofectamine TM 2000 (LF2000, Invitrogen) according to the manufacturer’s protocol. For silencing of SOCS3 or STAT5 gene expression, transfected cells were cultivated for 24 h .The cells were then collected for further experiments.

### 3.8. Statistical Analysis

Results were reported as mean ± SE. All the data were obtained from at least three independent experiments. Data statistics and individual differences among groups were analyzed using *t*-test by Sigma Plot 9.0 software, and differences were considered statistically signiﬁcant if *p* < 0.05 or *p* < 0.01.

## 4. Conclusions

SOCS3 inhibited β-casein gene expression and cell proliferation as an negative feedback-loop regulator of JAK2/STAT5 signaling pathway in DCMECs. Met can up-regulate milk protein synthesis by inhibition of expression of SOCS3. STAT5 is required for SOCS3 activation in DCMECs. We also noted a change in mTOR and SREBP-1c expression, due to activation and inhibition of SOCS3.
